# Shenzhi Jiannao formula ameliorates vascular dementia in vivo and in vitro by inhibition glutamate neurotoxicity via promoting clathrin-mediated endocytosis

**DOI:** 10.1186/s13020-021-00477-4

**Published:** 2021-07-28

**Authors:** Danfeng Tian, Yangyang Guo, Dandan Zhang, Qiang Gao, Ganlu Liu, Jingfeng Lin, Ze Chang, Yuchun Wang, Rui Su, Zhenyun Han

**Affiliations:** 1 Beijing University of Chinese Medicine, Beijing, 100029 China; 2 Department of Scientific Research, Beijing Hospital of Traditional Chinese Medicine, Capital Medical University, Beijing, 100010 China; 3 Shenzhen Hospital of Beijing University of Chinese Medicine (Longgang), No. 1 Dayun Road, Longgang District, Shenzhen, 518172 China

**Keywords:** Shenzhi Jiannao formula, Vascular dementia, Clathrin-mediated endocytosis, Glutamate, Neurotoxicity, Cognition recovery

## Abstract

**Background:**

Synaptic damage and glutamate excitotoxicity have been implicated in the pathogenesis of vascular dementia (VD). Clathrin, RAB5B and *N*-methyl-d-aspartic acid receptor 1 (NMDAR1) proteins play a vital role in endocytosis of synaptic vesicles in neurons and glutamate over accumulation. Previous researches have been confirmed that Shenzhi Jiannao (SZJN) formula has an anti-apoptotic and neuroprotective effect in VD, but the underlying mechanisms are still unclear. In this study, we aimed to explore the effect of SZJN formula on cognitive impairment and glutamate excitotoxicity via clathrin-mediated endocytosis (CME) in vivo and in vitro.

**Methods:**

SZJN formula consists of *Panax ginseng* C.A.Mey., *Anemarrhena asphodeloides* Bunge, and *Paeonia anomala* subsp. *veitchii* (Lynch) D.Y.Hong & K.Y.Pan. All herbs were prepared into granules. Both common carotid arteries were permanent occluded (2‐vessel occlusion, 2VO) in male Sprague Dawley (SD) rats to model VD. One day after operation, the rats began daily treatment with SZJN formula for 2 weeks. The neuroprotective effects of SZJN formula was subsequently assessed by the novel object recognition test, Morris water maze, hematoxylin–eosin (HE) staining and Nissl staining. Glutamate cytotoxicity was assessed by detecting cell viability and cell death of PC12 cells. Immunohistochemistry, immunofluorescence, Western blot, and quantitative real‐time PCR were used to detect the expression levels of clathrin, RAB5B, and NMDAR1.

**Results:**

Administration of SZJN formula effectively improved short-term memory and spatial memory. SZJN formula treatment significantly reduced hippocampal neuronal loss, and recovered the arrangement and morphology of neurons and Nissl bodies. Moreover, SZJN formula promoted the proliferation of PC12 cells and inhibited glutamate-induced cell death. The down-regulation of clathrin and RAB5B, as well as the upregulation of NMDAR1 in the brain induced by 2VO or glutamate was also notably reversed by SZJN formula at both the protein and mRNA levels, which may contribute to SZJN formula induced improved neurological function.

**Conclusions:**

Taken together, our findings provide evidence that the neuroprotective effects of SZJN formula in experimental VD maybe mediated through promoting the expression of clathrin-mediated endocytosis and reducing NMDARs‐associated glutamate excitotoxicity. SZJN formula serves as a promising alternative therapy and may be a useful herbal medicine for preventing progression of VD.

**Graphic abstract:**

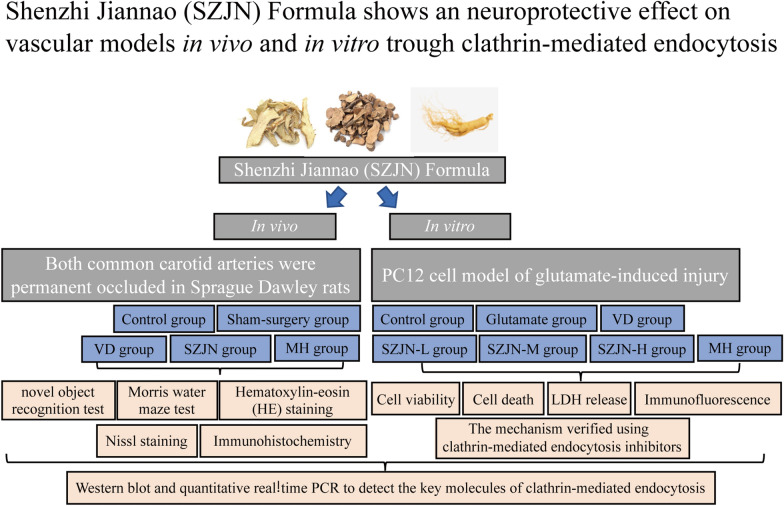

**Supplementary Information:**

The online version contains supplementary material available at 10.1186/s13020-021-00477-4.

## Introduction

Vascular dementia (VD) is the second most common type of senile dementia and maybe the main dementia type in East Asia [1]. Globally, there are about 17 million cases of dementia suffering from VD at an annual cost of up to $200 billion [2]. The pathogenesis of VD is closely related to glutamate-induced excitotoxicity and the impairment of glutamatergic vesicle trafficking [3, 4]. Glutamate is the most important excitatory neurotransmitter in the central nervous system which could regulate synaptic transmission, synaptic plasticity and cognitive abilities [5, 6]. Studies have confirmed that the excessive release of glutamate in the synaptic cleft and subsequent Ca^2+^ influx via NMDA-subtype glutamate receptors could lead to an intracellular cascade of cytotoxic events [7, 8]. The excessive activation of NMDA receptors evokes the impairment of synaptic plasticity, neuronal dysfunction and death [9, 10].

The endocytosis of synaptic vesicles, especially glutamatergic vesicle trafficking, is of great importance in the nervous system [11, 12]. It has been demonstrated that the dysfunction of vesicle signaling at the end of axons could lead to neurosynaptic damage, abnormal dendritic structures, and eventually neuronal apoptosis [13]. Clathrin-mediated endocytosis (CME) is the major route for receptor internalization at the plasma membrane, in which clathrin as well as RAB5B are two key molecules responsible for the formation of synaptic vesicles, endocytosis, synaptic recycling, and release of neurotransmitter vesicles [14–16]. Additionally, the temporal cortex samples from VD patients showed significantly lower levels of synaptic-related proteins, especially the expression level of clathrin [17]. Although the exact mechanism of glutamate transmission remains unknown, evidence has been proved that CME plays an important role in internalization of NMDA receptors [18, 19]. The injury of CME process would delay the clearance of extracellular glutamate and cause glutamate toxicity in the brain. Hence, strategies to promote clathrin-mediated endocytosis of NMDA receptors to reduce glutamate neurotoxicity and recovery of synaptic plasticity may be of significant interest for the development of future therapies in VD.

SZJN formula, an important Chinese prescription, has been used for the treatment of chronic cerebral hypoperfusion and dementia. It is composed of *Panax ginseng* C.A.Mey., *Anemarrhena asphodeloides* Bunge, and *Paeonia anomala* subsp. *veitchii* (Lynch) D.Y.Hong & K.Y.Pan, which have been officially recorded in the Chinese Pharmacopoeia 2020. The optimal ratio of these three herbs is 1:3:3 [20]. Pharmacological studies have shown that *Panax ginseng* C.A.Mey. has the function of regulating neurotransmitters levels, inhibiting hippocampal neuron apoptosis, and attenuating cerebral ischemia injury and cognitive deficits [21–24]. *Anemarrhena asphodeloides* Bunge has been verified to have cholinesterase inhibitory activity, and it could inhibit neuroinflammation and remove oxygen free radicals to improve learning and memory abilities [25, 26]. *Paeoniae Radix Rubra* has also been demonstrated to have inhibitory effects on thrombus formation, as well as anti-inflammatory and antioxidative properties to reduce neurotoxic injury [27–29]. In our previous studies, we showed that SZJN formula has potent neuroprotective effects through regulation of neurotransmitters levels, inhibition of apoptotic cell death, and improving learning and memory deficits in VD models [30–32]. However, it is still not clear how SZJN formula inhibits glutamate neurotoxicity in VD models.

In this study, we observed the effect of SZJN formula on clathrin-mediated endocytosis and the toxic accumulation of glutamate in vivo and in vitro, and further explored its possible mechanism of learning and memory function improvement.

## Materials and methods

### Animals

Adult male Sprague Dawley (SD) rats (250 ± 20 g) were purchased from Beijing Vital River Laboratory Animal Technology Co., Ltd (certificate number: SCXK (Beijing) 2016-0006). The animals were housed with temperature (22 ± 1 °C), humidity (50 ± 5%), and light (12-h light/dark cycle) control with free access to water and food. All experiments were conducted after being approved by the Institutional Animal Ethical Care and Use Committee of the Beijing University of Chinese Medicine (Approval ID: BUCM-4-2017121725-4025).

### Animal surgical procedure

VD model was established by permanent bilateral common carotid artery occlusion (2VO) as described before [33]. Briefly, rats were anesthetized by intraperitoneal injection of 2% sodium pentobarbital (45 mg/kg). The neck of the rats was disinfected, and an incision was made to expose and separate the bilateral common carotid arteries along the cervical anterior median. Then, the bilateral blood vessels were ligated with 5–0 silk thread. In the sham-surgery group, the same surgery was performed with the exception of arterial ligation. The rats’ body temperature was maintained at about 37 ± 1 °C during surgery.

### Experimental design

SZJN formula is composed of 3 g *Panax ginseng* C.A.Mey., 9 g *Anemarrhena asphodeloides* Bunge, and 9 g *Paeonia anomala* subsp. *veitchii* (Lynch) D.Y.Hong & K.Y.Pan. All herbs were pharmacopoeia-grade and prepared into granules by Beijing Tcmages Pharmaceutical Co., Ltd. (Beijing, China). Details of herbal materials are listed in Additional file 1: Fig. S1. The material basis of SZJN formula was determined using high-performance liquid chromatography (HPLC) and infrared spectrum (IS) analysis for monitoring the quality control purposes (Additional file 2: Fig. S2). Ginsenoside Rg1, ginsenoside Re, ginsenoside Rb1, Mangiferin, and paeoniflorin (the purities of all standards were higher than 98% by high-performance liquid chromatography analysis) were purchased from Beijing Solarbio Biotech Co., Ltd. (Beijing, China). The granules were suspended with distilled water to a final concentration of 38 g/L.

**Fig. 1:**
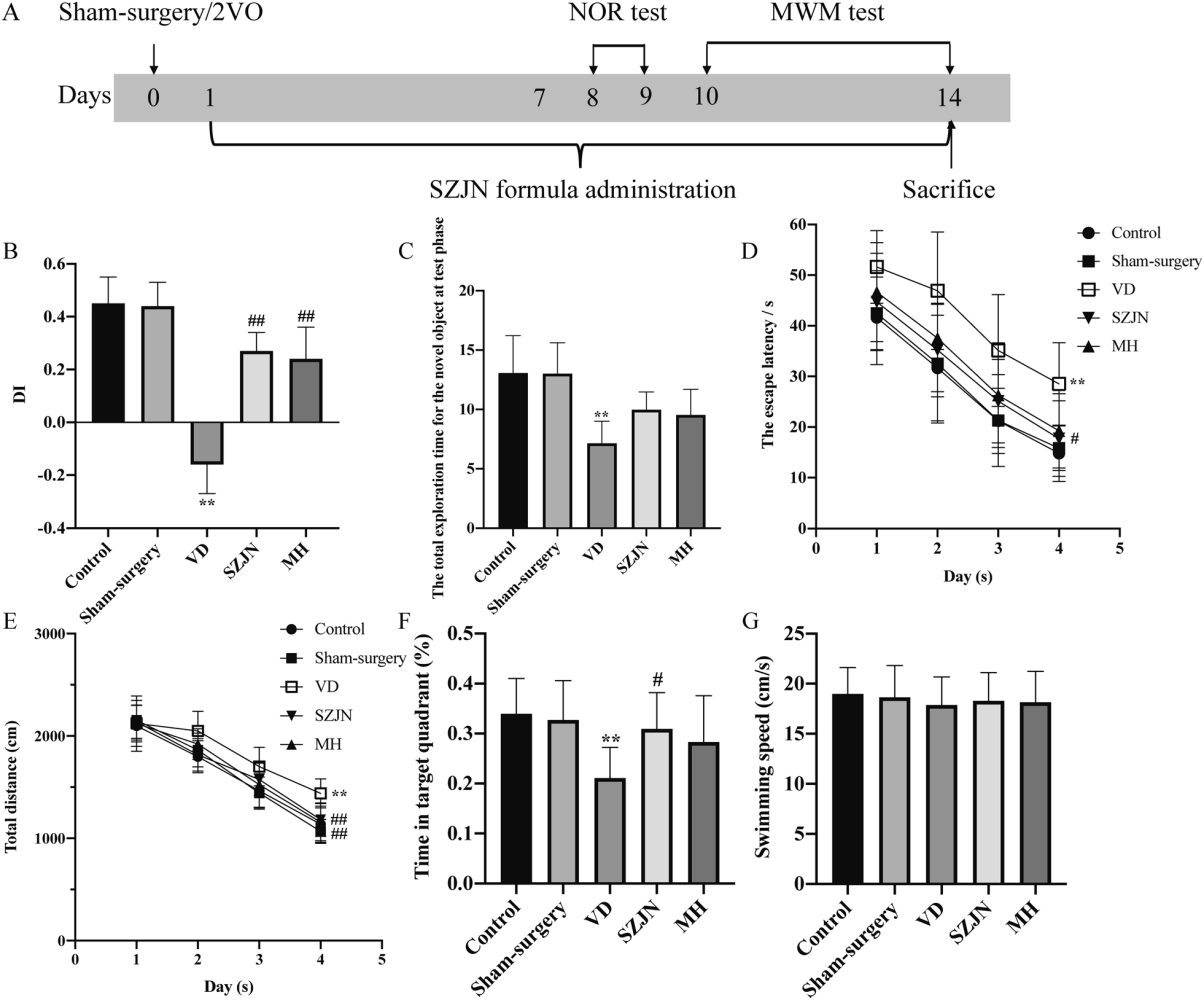
SZJN formula ameliorated cognitive deficits and improved memory abilities in VD rats. **A** Schematic timeline of the animal study. **B** Discrimination index (DI) of NOR test. **C** The total exploration time for the novel object at test phase. **D** The escape latency and **E** total distance for reaching the hidden platform during 4 days in the probe navigation trial. **F** The time spent in the target quadrant and **G** swimming speed in the spatial probe trial. Values were expressed as means ± SD (n = 12). ^*^P < 0.05, ^**^P < 0.01 versus sham-surgery group. ^#^*P* < 0.05, ^##^*P* < 0.01 versus VD group

Rats were randomly divided into control group, sham-surgery group (n = 12), VD group (n = 12), SZJN group (n = 12), and memantine hydrochloride (MH, H.Lundbeck A/S, Denmark) group (n = 12). Rats in the SZJN group and MH group were intragastrically administered with SZJN formula (dissolved with distilled water, 0.2 g/kg), and memantine hydrochloride (dissolved with distilled water, 2.1 mg/kg) one day after surgery for 14 days, respectively [31]. In the control group, sham-surgery group and VD group, rats were administered with equivalent distilled water intragastrically once per day for 14 days. Figure 1A presents a schematic timeline of experiments.

### PC12 cell culture and experimental design

Differentiated PC12 cells (Procell Life Science & Technology Co., Ltd., Wuhan, China) were maintained in RPMI 1640 (Procell) supplemented with 10% (v/v) fetal bovine serum (Procell)) and an antibiotic mixture of penicillin and streptomycin (1%, Procell) in a humidified incubator at 37 °C and 5% CO_2_. When these cells reached 70%–80% confluence, they were trypsinized and sub cultured. After seeding onto poly-L-lysine-coated 96- or 6-well plates at 3 $\times {10}^{4}$ cells/well for 24 h, the cells were exposed to glutamate (final concentration: 22.5 mM, Sigma, USA) and incubated in the presence or absence of various concentrations of SZJN formula (final concentrations: 0.05, 0.1 and 0.2 mg/mL) and memantine hydrochloride (MH, 10 μM) for 24 h. The control cells were not administered any test agent or glutamate as the vehicle control. The glutamate-exposed cells were treated with glutamate for 24 h alone. The concentrations of SZJN formula in this experiment were selected according to our preliminary experiment (Additional file 3: Fig. S3). For inhibition of endocytosis, chlorpromazine (CPZ, 10 μM, Sigma) and Pitstop 2 (20 μM, Abcam) were pre-treated to the culture medium at 37 °C for 30 min and 10 min, respectively. All operations were replicated three times under each treatment condition for each experiment.

### Cell death and morphology changes

To observe cell death and morphology changes during intervention process, the live-cell Essen Bioscience IncuCyte imaging system was used. Briefly, PC12 cells cultured by different concentrations of test agents were exposed to 5% YOYO™-1 lodide solution, a cell impermeable dye that only enters cells with compromised membranes. The samples were observed under the IncuCyte imaging system at 10× magnification, which records both phase-contrast as well as fluorescent images over time.

### Cell viability analysis

Cell viability was assessed using a cell counting kit 8 (CCK8, Dojindo Laboratories, Japan). Briefly, PC12 cells were incubated with 10 μL CCK‐8 solution at 37 °C in the dark for 1–2 h. Absorbance was measured at 450 nm using a microplate reader (Multiskan GO, Thermo Fisher Scientific, USA).

### Determination of lactate dehydrogenase (LDH) release

After treatment, the cell culture supernatant (80 μL) was collected and added to 96-well plates for lactate dehydrogenase (LDH) determination using the LDH assay kit (Servicebio, Wuhan, China) following the manufacturer’s instructions. Absorbance was measured at 490 nm using a microplate reader (Multiskan GO, Thermo Fisher Scientific, USA).

### Novel object recognition (NOR) test

NOR test was performed on the 8th day of treatment as previously described [34]. During the training period, the exploration time for two identical objects was recorded within 5 min. One day later, one object was changed to a different color and shape but with the same size. The exploration time for the new object and the familiar one was recorded within 5 min. Rats were evaluated for their memory by expressing a preference for exploring the novel object [35]. Preference for the novel object was expressed as a discrimination index (DI, equation below).

$${\text{DI}} = \frac{{{\text{time exploring the novel object}} - {\text{time exploring the familiar object }}}}{{{\text{time exploring the novel object}} + {\text{time exploring the familiar object}}}}$$

### Morris water maze (MWM) test

To assess spatial learning and memory, MWM test was performed on the 10th day of treatment according to a procedure described with some modifications [36]. The circular water maze pool was 120 cm in diameter and divided into 4 quadrants. Each rat was allowed to search for the platform within 60 s. When the rats failed to locate the submerged platform, they were manually directed toward the platform and allowed to remain on it for 10 s. Each rat was trained 4 times a day for 4 consecutive days. On the 5th day, a spatial probe trail was conducted by removing the platform from the pool. The rats were monitored to swim freely for 60 s. The escape latency and total swimming distance in the training trail, percentage time spent in the target quadrant and swimming speed in the probe trail were recorded. All rats were kept from a motor impairment.

### Histological and pathological assessment

Rats were anesthetized using 2% sodium pentobarbital administered intraperitoneally (45 mg/kg) and transcardially perfused with normal saline (50–100 mL) at 37 °C and then perfused with 4% paraformaldehyde (100 mL) for 30 min. The brain was immersion fixed in 4% paraformaldehyde for 48 h after which the brains were embedded in paraffin. A series of adjacent 3 μm thick coronal sections were cut and employed for various staining. Additional hippocampal tissues were frozen in liquid nitrogen and stored at − 80 °C for further analysis. For hematoxylin–eosin (H&E) staining and Nissl staining, slices were dehydrated with EtOH (70%, 95%, 100%, 5-min each), until xylene (5-min), followed by staining using hematoxylin–eosin as well as toluidine blue, respectively. Histopathological changes in the hippocampi were observed using a light microscope (Olympus BX53, Japan) and counted at 200× magnification.

### Immunohistochemistry

Brain coronal sections were incubated for 1 h at room temperature with 10% goat serum then overnight at 4 °C with primary antibodies to clathrin, RAB5B and NMDAR1 (Additional file 4: Table S1). After incubation with secondary antibodies for 50 min, immunostaining was detected with 3,3′-diaminobenzidine kit (DAB, G1211, Servicebio). Image pro plus 6.0 image analysis software (Media Cybernetics, Rockville, MD, USA) was used for data analysis. The Area and Integral Optical Density (IOD) of the positive regions were imaged and the relative protein expression levels were calculated as the mean optical density (IOD/Area).

### Immunofluorescence

PC12 cells were rinsed with PBS and fixed with 95% ethanol or 4% paraformaldehyde. After blocked with 3% BSA for 1 h at 37 °C, cells were incubated with primary antibodies (Additional file 4: Table S1) for 2 h at 37 °C. Subsequently, cells were washed and incubated with secondary antibodies in the dark for 1 h at 37 °C, followed by staining with 20 μg/ mL Hoechst 33342 (Sigma-Aldrich) for 15 min. A IN Cell Analyzer high connotation cell imaging analysis system (2500HS, GE, USA) was used the observation.

### Western blot analysis

Minute™ Plasma Membrane Protein Isolation and Cell Fractionation Kit (Invent Biotechnologies, USA) was used to isolate and extract cytomembrane and cytoplasmic proteins according to the manufacturer’s instruction. The hippocampal tissue was added with 200 μL buffer A and grinded with a grinding rod for about 1 min before adding 300 μL of buffer A. After incubation on ice for 5 min, gradient centrifugation was performed. The extracted protein of cells or tissues was determined for concentration with a BCA kit (Servicebio). Equal amounts of protein were electrophoresed on 10% polyacrylamide gels and then transferred onto a polyvinylidene fluoride (PVDF) membrane. After blocking with 5% skim milk for 1 h, the membranes were incubated overnight at 4 °C with primary antibodies directed against clathrin, RAB5B, NMDAR1, and β-actin (Additional file 4: Table S1). The membranes were incubated with secondary antibodies at room temperature for 1 h and visualized using an ECL substrate (WBKLS0100, Millipore). FlourChem software (Alpha Innotech, FlourChem) was used to analyze the ratio of the gray value of each band to the β-actin band for relative expression levels. All samples were analyzed independently and in triplicate.

### Quantitative real-time PCR

Total RNA was extracted from tissue samples and cells using a HiPure Total RNA Mini Kit (R4111-02, Magen) following the manufacturer instructions. After reverse transcription using the RevertAid First Strand cDNA Synthesis Kit (Thermo Fisher Scientific, USA), cDNA was used as the template for quantitative RT-PCR and performed using the real-time fluorescence quantitative PCR instrument (CFX96, Bio-Rad). The PCR cycle was as follows: 95 °C pre-denaturation for 10 min, 95 °C denaturation for 15 s, 60 °C annealing for 1 min, with a total of 40 cycles. β-actin was designated as the internal reference gene and the relative expression level of target genes was calculated by the ${2}^{-\Delta \Delta \mathrm{CT}}$ method. All samples and assays were performed in triplicate. Primers (Sangon Biotech, Shanghai, China) for clathrin, NMDAR1, and β-actin are listed in Additional file 4: Table S2.

### Statistical analysis

SPSS 25.0 software was used for data analysis. Data were expressed as mean ± SD. MWM data were analyzed using two-way repeated measure ANOVA. For the others were analyzed by one-way ANOVA followed by post hoc test (Tukey's tests). If the variance of statistics was uneven, a Nonparametric test was performed. *P* values < 0.05 was considered statistically significant.

## Results

### SZJN formula ameliorated cognitive deficits in VD rats

**Fig. 2:**
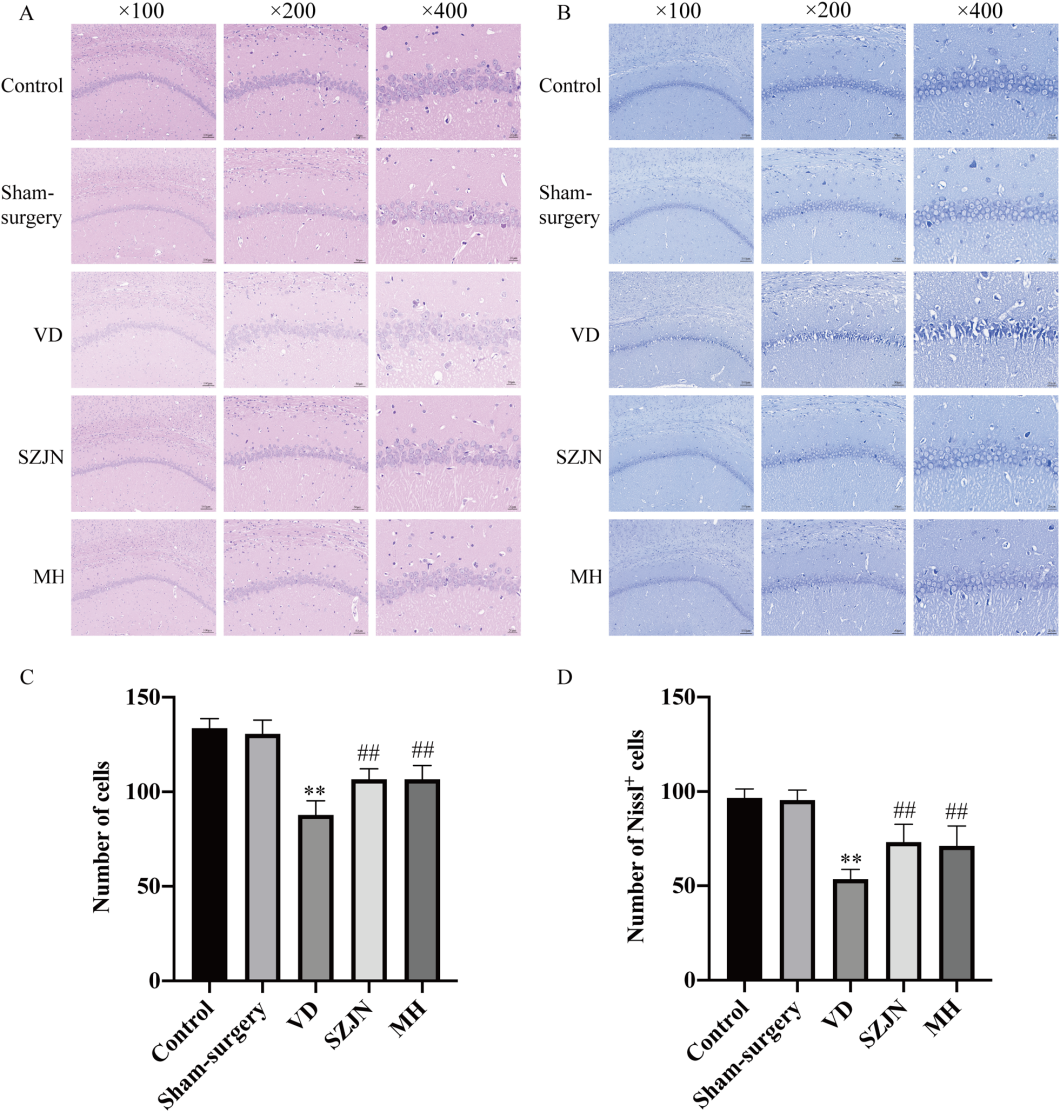
SZJN formula inhibited hippocampal neuronal damage and loss in VD rats. Representative images and quantitative graph of **A**, **C** H&E staining and **B**, **D** Nissl staining in hippocampal CA1 region were performed as described in Methods (×100, ×200, ×400). Values were expressed as means ± SD (n = 6). ^*^P < 0.05, ^**^P < 0.01 versus sham-surgery group. ^#^*P* < 0.05, ^##^*P* < 0.01 versus VD group

One week after the administration of SZJN formula, animals were tested in a series of behavioral experiments including NOR test and MWM test cognition and memory ability. In 2 days of the NOR test, the total exploration time for the novel object at test phase and the discrimination index was decreased in the VD group compared with the sham-surgery group (*P* < 0.01, n = 12). In contrast, VD rats treated with SZJN formula or MH exhibited significantly higher discrimination index indicating improved short-term memory, in comparison to VD model rats (*P* < 0.01, n = 12). No significant difference was found between the control and sham-surgery groups (*P* > 0.05, n = 12; Fig. 1B, C). For the escape latency and total swimming distance in the MWM test, there was a significant effect over time (F[2.538, 139.6] = 104.1, *P* < 0.0001; F[2.757, 151.6] = 362.4, *P* < 0.0001) and also a significant effect for treatment (F[4, 55] = 15.36, *P* < 0.0001; F[4, 55] = 7.244, *P* < 0.0001). The interaction of treatment and time in the escape latency was not significant (F[12, 165] = 0.1675, *P* > 0.05), and the interaction of treatment and time in the total swimming distance was significant (F[12, 165]  = 2.136, *P* < 0.05). The escape latency in the VD group was significantly longer than that in the sham-surgery group on day 1, 2, 3, and 4 (*P* < 0.01, n = 12; Fig. 1D), during the probe navigation training. On the contrary, the escape latency of SZJN and MH groups was significantly shorter compared with the VD group (*P* < 0.01, *P* < 0.05, n = 12; Fig. 2D). The total distance in SZJN and MH groups decreased significantly, compared with VD group (P < 0.01, n = 12; Fig. 1E). After the hidden platform was removed, the time spent in the target quadrant of the SZJN group was significantly higher than that of the VD group (*P* < 0.05, n = 12; Fig. 1F). Likewise, MH treatment yielded the similar improvements but there was no significant difference compared to the model group (*P* > 0.05, n = 12; Fig. 2F). In addition, the swimming speed was not significantly different between each group (*P* > 0.05, n = 12; Fig. 1G), indicating no motor function impairment in all the groups. These data indicate that both the SZJN formula and MH improves cognitive function and memory in VD rats.

### SZJN formula administration alleviated neuronal damage and death in the hippocampus of VD rats

H&E and Nissl staining was performed to determine the neuroprotective effects of SZJN formula on the hippocampal CA1 region of brain tissues. As shown in Fig. 2A, neurons were arranged tightly and orderly with clear nuclei in the hippocampus of the control and sham-surgery group (n = 6). However, obvious pathological abnormalities with scattered neurons arrangement, neurons shrunken and loss, or dark color staining were observed significantly in the VD group (*P* < 0.01, n = 6; Fig. 2A and C). In comparison, both SZJN formula and MH alleviated these histopathological alterations (n = 6; Fig. 2A). Additionally, SZJN formula or MH treatment for 2 weeks caused remarkable reduction in neurons loss in the hippocampal CA1 of rats, compared to the VD group (*P* < 0.01, n = 6; Fig. 2C).

For Nissl staining, neurons exhibited a normal morphology with distinct round or oval nuclei and nucleoli, and clear Nissl bodies in the cytoplasm in the control and sham-surgery groups (n = 6; Fig. 2B). In comparison, a great number of apoptotic neurons, with Nissl body fragmentation, cell loss and karyorrhexis were observed in the VD group (n = 6). The number of Nissl‐positive cells significantly decreased in the hippocampal CA1 region of VD rats, compared to the sham-surgery group (*P* < 0.01, Fig. 2D). Compared with the VD group, administration of SZJN formula and MH clearly improved the morphology and arrangement of neurons, as well as markedly reversed 2VO‐induced neuronal loss in the CA1 region (*P* < 0.01, *P* < 0.05, n = 6; Fig. 2B and D).

### SZJN increased clathrin and RAB5B expression, whereas reduced cytomembrane NMDAR1 expression in the hippocampus of VD rats

**Fig. 3:**
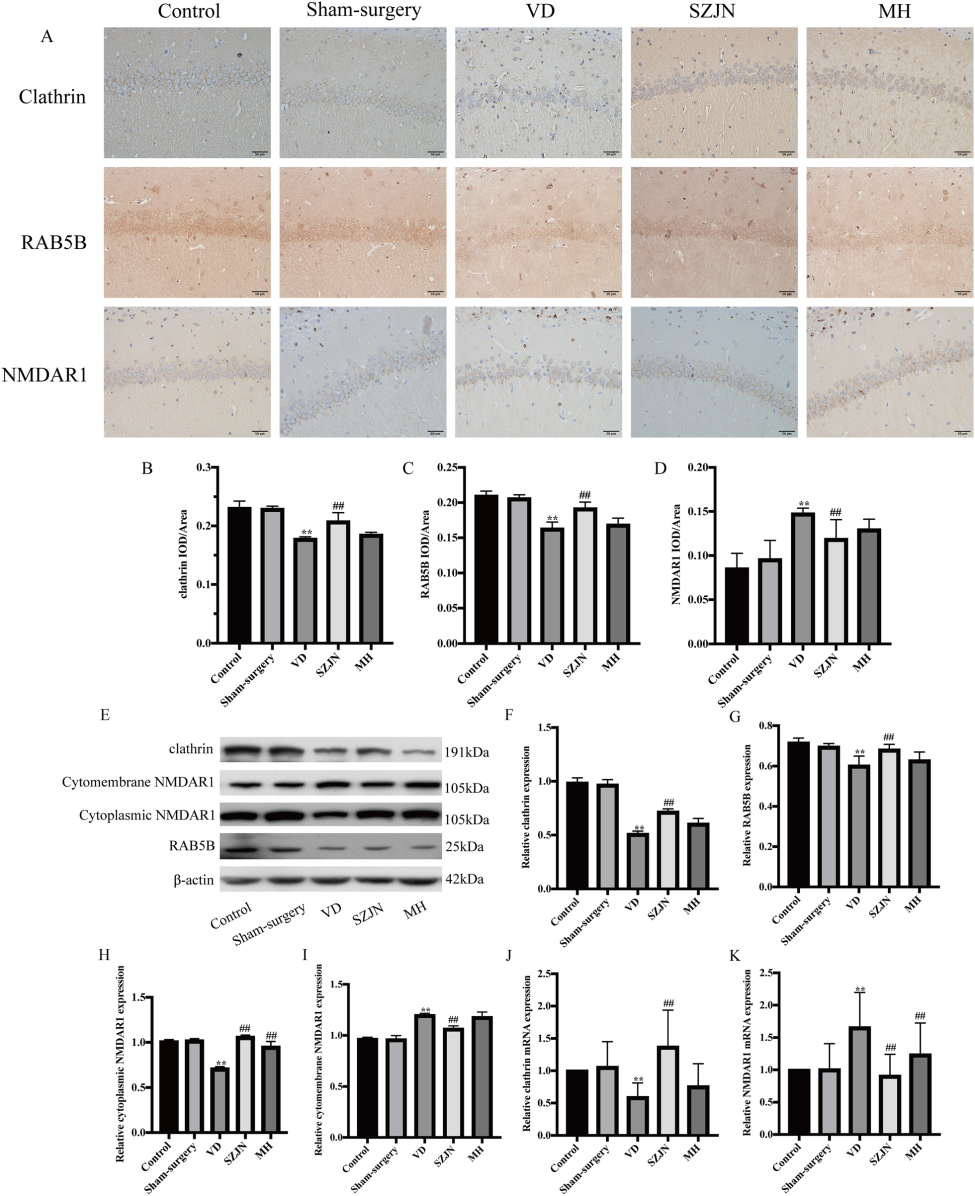
SZJN increased clathrin and RAB5B expression, whereas reduced cytomembrane NMDAR1 expression in the Hippocampus of VD Rats. **A** Immunohistochemical analysis of clathrin, RAB5B, and NMDAR1 expression in hippocampal CA1 regions (×400). Quantitative analysis showed the expression of **B** clathrin, **C** RAB5B, and **D** NMDAR1 in hippocampal CA1 regions. **E** Western blot was performed to detect the levels of clathrin, RAB5B, cytoplasmic NMDAR1, and cytomembrane NMDAR1 in the hippocampus of VD rats. Quantitative analysis of **F** clathrin, **G** RAB5B, **H** cytoplasmic NMDAR1, and **I** cytomembrane NMDAR1 expression. The densities of the bands were normalized with respect to the values of β-actin. qRT-PCR analyzed **J** clathrin mRNA and **K** NMDAR1 mRNA expression levels. Values were expressed as means ± SD (n = 6). ^*^P < 0.05, ^**^P < 0.01 versus sham-surgery group. ^#^*P* < 0.05, ^##^*P* < 0.01 versus VD group

We evaluated whether SZJN formula could inhibit glutamate toxicity and promote the CME process associated with upregulating clathrin and RAB5B, and downregulating NMDAR1 in the hippocampus. Immunohistochemical staining of the hippocampus illustrated that the expressions of clathrin and RAB5B were decreased significantly in VD rats (*P* < 0.01, n = 6), while increased in the SZJN group (*P* < 0.01, n = 6; Fig. 3A–C). The activation of NMDAR1 was significantly increased in VD rats (*P* < 0.01), while decreased in the SZJN group (*P* < 0.01, n = 6; Fig. 3A and D). Western blot analysis showed similar results as the immunohistochemistry. The expressions of clathrin, RAB5B and cytoplasmic NMDAR1 in the hippocampus were increased dramatically in the VD group compared with the sham-surgery group (*P* < 0.01, n = 6; Fig. 3E–H). However, administration with SZJN formula showed a remarkable increase of clathrin, RAB5B and cytoplasmic NMDAR1 expression compared with the VD group (*P* < 0.01, n = 6). The expression of cytomembrane NMDAR1 was significantly increased in VD rats (*P* < 0.01, n = 6), while decreased in the SZJN group (*P* < 0.01, n = 6; Fig. 3E and I).

qRT-PCR showed that the mRNA level of clathrin was reduced in the VD group compared with the sham-surgery group (*P* < 0.01, n = 6). In contrast, rats in the SZJN group showed obviously increased mRNA level of clathrin in comparison to those in the VD group (*P* < 0.01, n = 6; Fig. 3J). Moreover, the mRNA expression of NMDAR1 increased in the hippocampus of VD rats compared to the sham‐surgery rats (*P* < 0.01, n = 6). However, SZJN formula decreased the expression of NMDAR1 notably in the hippocampus (*P* < 0.01, n = 6; Fig. 3K).

### SZJN formula rescued the VD cell model from glutamate-induced PC12 cell damage

**Fig. 4:**
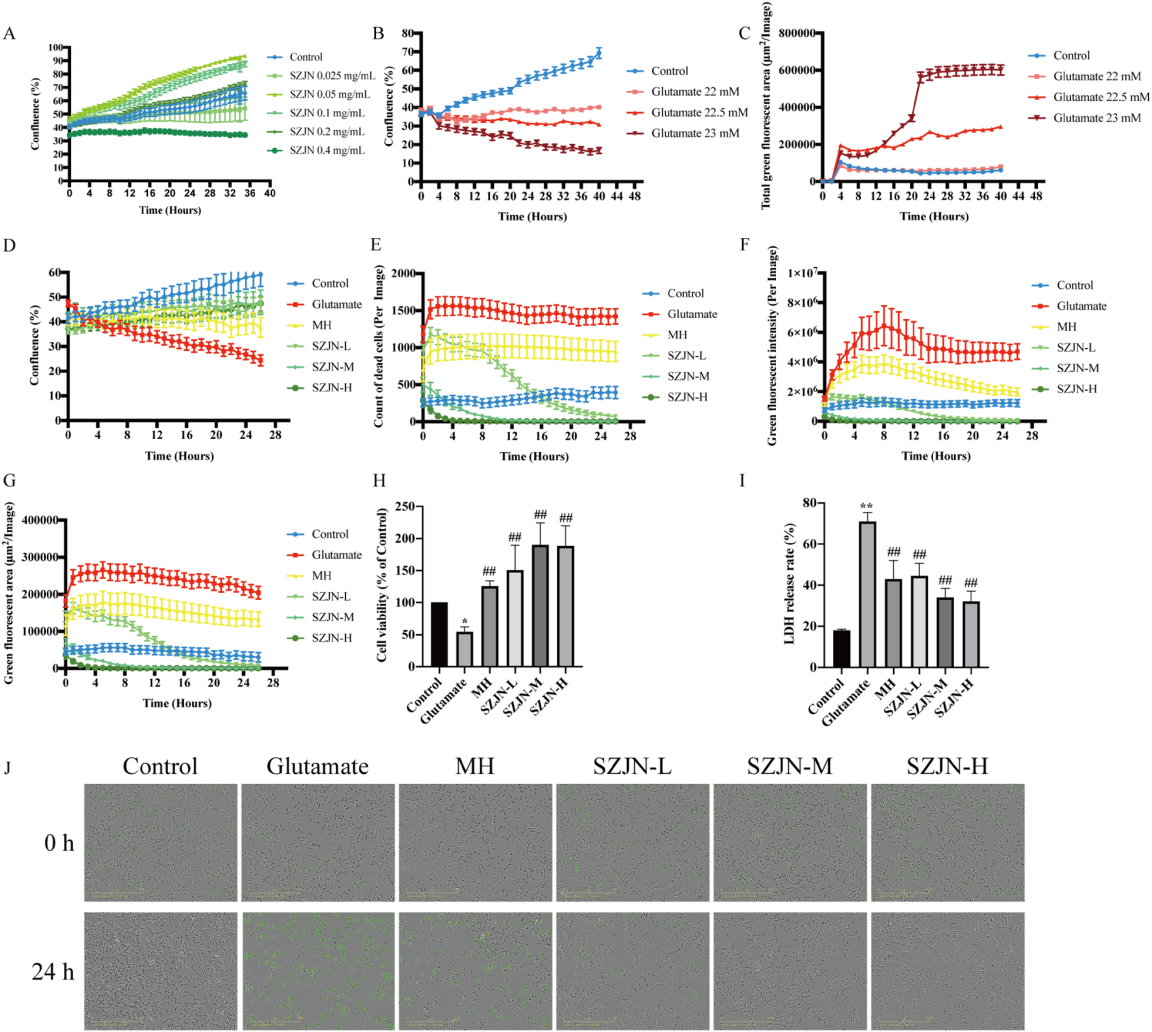
Effects of SZJN formula on glutamate-induced cytotoxicity to PC12 cells. **A** Effects of SZJN formula (0.025, 0.05, 0.1, 0.2, and 0.4 mg/mL) on basal growth of PC12 cells. **B** Effects of glutamate (22, 22.5 and 23 mM) on PC12 cell viability. **C** Effects of glutamate (22, 22.5 and 23 mM) on the area of total green fluorescence. **D** Effects of SZJN formula on cell viability, **E** number of dead cells, **F** green fluorescent intensity, and **G** green fluorescent area in glutamate-exposed PC12 cells. Following treatment of the cells with various concentrations of SZJN formula (0.05, 0.1, and 0.2 mg/mL) for 24 h and exposure to 22.5 mM of glutamate for 24 h. **H** Effects of SZJN formula on cell viability using CCK8 assay. **I** Effects of SZJN formula on LDH release in PC12 cells. **J** Effects of SZJN formula in glutamate-induced cell morphological changes. SZJN-L: 0.05 mg/mL, SZJN-M: 0.1 mg/mL, SZJN-H: 0.2 mg/mL. Values were expressed as means ± SD (n = 4). ^*^P < 0.05, ^**^P < 0.01 as compared with the control group. ^#^*P* < 0.05, ^##^*P* < 0.01 as compared with the glutamate group

To observe the glutamate‐induced cytotoxicity and protective effects of SZJN formula on glutamate-induced neurotoxicity, the cell death, viability and LDH release was assessed using the IncuCyte imaging system, CCK8 and LDH cytotoxicity assay kit. The results (Fig. 4A) revealed that compared with the control group, the viability of the PC12 cells treated with SZJN formula alone with the concentrations of 0.05, 0.1, and 0.2 mg/mL was significantly increased, whereas SZJN formula with 0.025 and 0.4 mg/mL did not show the significance of promoting cell proliferation. Among the effective concentrations, 0.05 and 0.1 mg/mL of SZJN formula showed the best enhancing cell viability effect. Thus, SZJN formula at the concentrations of 0.05, 0.1, and 0.2 mg/mL was selected for use in the subsequent experiments. Then the concentration-dependent response of glutamate-induced cytotoxicity was determined. As shown in Fig. 4B, PC12 cell viability was slightly enhanced by glutamate with 22 mM, and inhibited by glutamate with 22.5 and 23 mM markedly. Besides, cells cultured with 22 mM glutamate did not show obvious green fluorescent area, whereas 22.5 mM glutamate showed 3 times and 23 mM glutamate showed 6 times of that in the control group by 24 hpt (Fig. 4C). Thus, glutamate at the concentration of 22.5 mM was selected for use in the subsequent experiments, combined with the screening results in Additional file 3: Fig. S3.

As shown in Fig. 4D, stimulation with glutamate alone resulted in a significant decrease in cell viability as compared with the control group by 24 hpt. The number of dead cells and green fluorescent intensity in the glutamate group were about 3 times of that in the control group by 24 hpt (Fig. 4E and F). Similarly, the green fluorescent area in the glutamate group increased by 0 hpt and increased to over 250,000/μm^2^ per image by 24 hpt (Fig. 4G). On the contrary, treatment with SZJN formula in the presence of glutamate exhibited a significant decrease of glutamate-induced toxicity in a concentration under 0.05, 0.1, and 0.2 mg/mL of SZJN formula. SZJN formula could reduce the number of dead cells, green fluorescent intensity and area, as well as recovered cell viability (Fig. 4D–G). Among the effective concentrations, 0.1 and 0.2 mg/mL of SZJN formula showed the best neuroprotective effect, as shown in Fig. 4E–G. CCK-8 cell viability assay showed that 0.05, 0.1, and 0.2 mg/mL SZJN formula and 10 μM MH could significantly increase the cell viability, while glutamate (22.5 mM) exhibited the significant cytotoxicity (*P* < 0.01, Fig. 4H). Moreover, the LDH release rate in the glutamate group was significantly higher than that in the control group (*P* < 0.01). SZJN formula and MH decreased the LDH release level evidently, indicating that they could inhibit the glutamate toxicity effectively (*P* < 0.01, Fig. 4I). Representative fluorescent images of cell morphological changes at 0 and 24 hpt are shown in Fig. 4J.

### SZJN formula enhanced clathrin and RAB5B expressions and reduced NMDAR1 expression in glutamate-induced PC12 cells

**Fig. 5:**
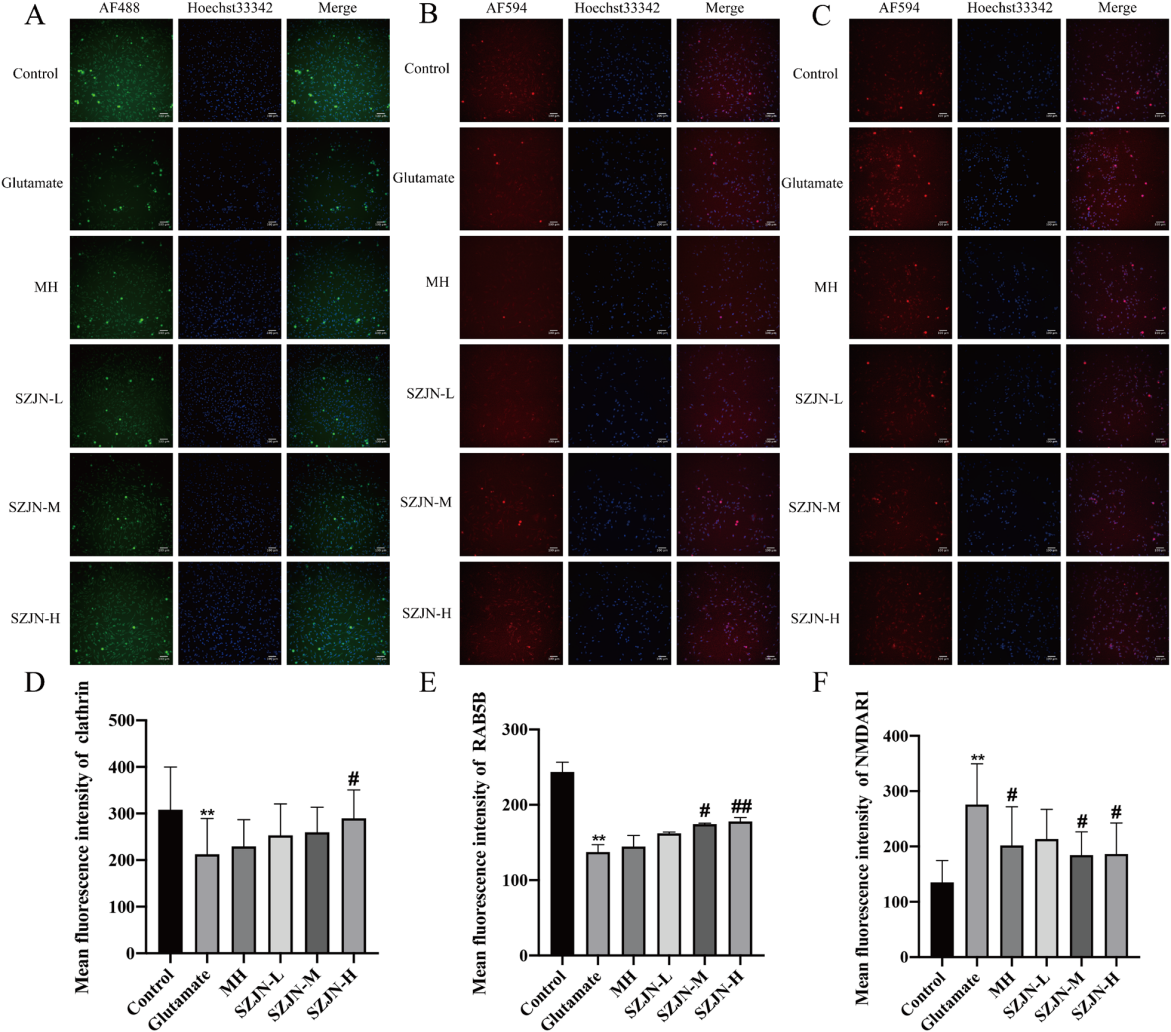
Effects of SZJN formula on the expression of clathrin, RAB5B, and NMDAR1 by using immunofluorescence. Representative photomicrographs of **A** clathrin (green, AF488), **B** RAB5B (red, AF594), and **C** NMDAR1 (red, AF594) immunofluorescence are shown. Nuclei were stained with Hoechst 33342 (blue). The graph show the mean fluorescence intensity of **D** clathrin, **E** RAB5B, and **F** NMDAR1 in PC12 cells. SZJN-L: 0.05 mg/mL, SZJN-M: 0.1 mg/mL, SZJN-H: 0.2 mg/mL. Values were expressed as means ± SD (n = 3). ^*^P < 0.05, ^**^P < 0.01 as compared with the control group. ^#^*P* < 0.05, ^##^*P* < 0.01 as compared with the glutamate group

To further explore whether the clathrin mediated glutamate synaptic vesicle transport was also regulated by SZJN formula in vitro, the protein and mRNA levels of clathrin, RAB5B, and NMDAR1 in glutamate induced PC12 cells were investigated. Immunofluorescence assay showed that the expressions of clathrin and RAB5B proteins were much lower and NMDAR1 expression was higher after treatment with glutamate (*P* < 0.01). After treatment with SZJN formula for 24 h, clathrin and RAB5B protein expressions were enhanced, as well as NMDAR1 expression was declined compared with the glutamate group (*P* < 0.05, Fig. 5A–F).

**Fig. 6:**
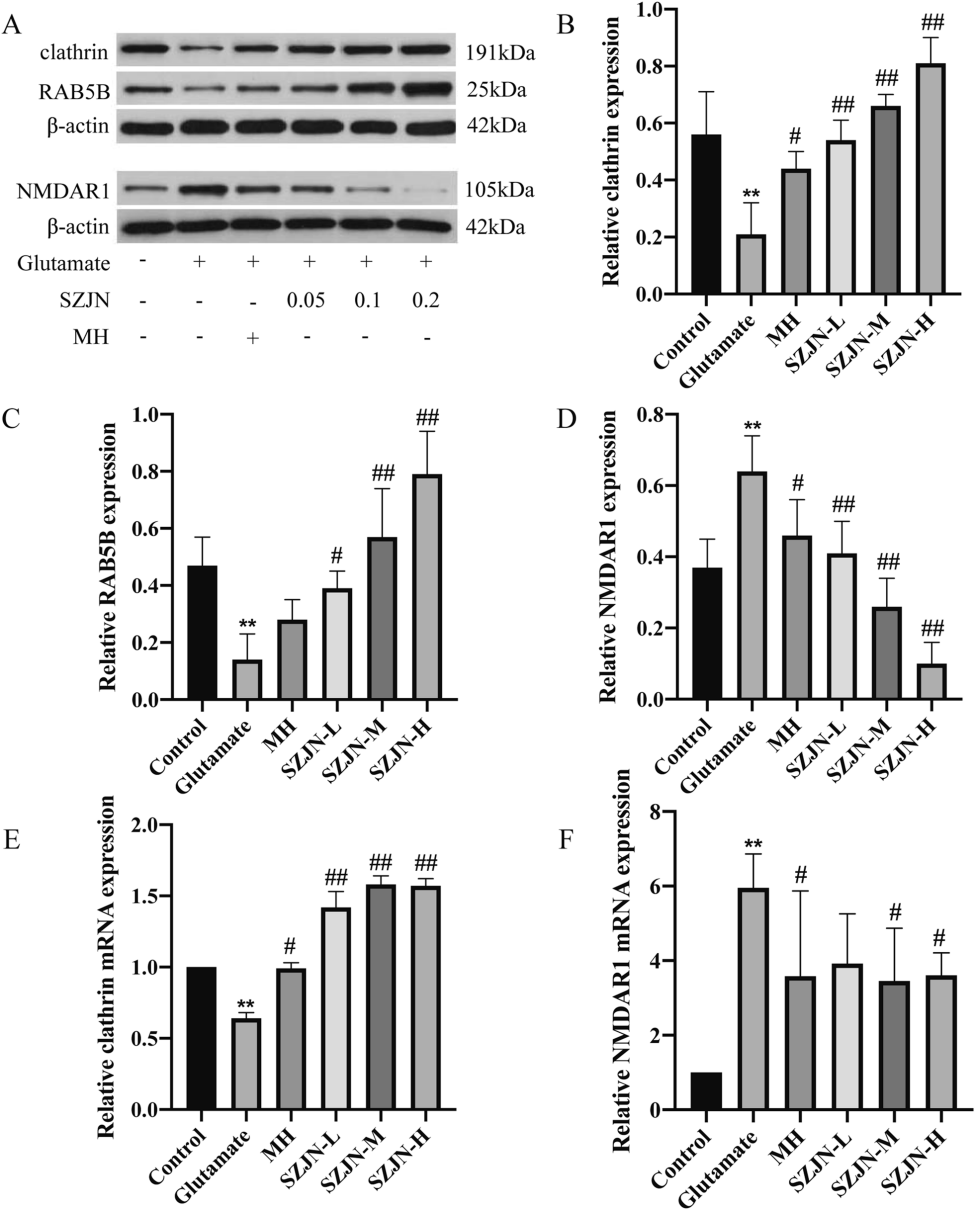
Effects of SZJN formula on the protein and mRNA expressions of clathrin, RAB5B, and NMDAR1. **A** Western blot was performed to detect the protein levels of clathrin, RAB5B, and NMDAR1 in glutamate treated PC12 cells. Quantitative analysis of **B** clathrin, **C** RAB5B, and **D** NMDAR1 expressions. β-Actin was used as the internal control. qRT-PCR analyzed **E** clathrin mRNA and **F** NMDAR1 mRNA expression levels. SZJN-L: 0.05 mg/mL, SZJN-M: 0.1 mg/mL, SZJN-H: 0.2 mg/mL. Values were expressed as means ± SD (n = 3). ^*^P < 0.05, ^**^P < 0.01 as compared with the control group. ^#^*P* < 0.05, ^##^*P* < 0.01 as compared with the glutamate group

Western blot analysis showed the similar results with Immunofluorescence. The expression of clathrin and RAB5B were markedly decreased (*P* < 0.01), but significantly elevated with SZJN formula treatment in a dose-dependent manner (*P* < 0.01, Fig. 6A–C). Meanwhile, after glutamate exposure, the expression of NMDAR1 was drastically elevated in the glutamate group as compared with the control group (*P* < 0.01), but down-regulated by SZJN formula in a dose-dependent manner (*P* < 0.01, Fig. 6A and D). In addition, clathrin and NMDAR1 levels were also reversed by MH treatment (*P* < 0.05), but RAB5B level had no significant difference (*P* > 0.05, Fig. 6A–D). Moreover, qRT-PCR demonstrated that the mRNA level of clathrin decreased and NMDAR1 mRNA level increased in glutamate group (*P* < 0.01). SZJN formula not only enhanced clathrin gene expression (*P* < 0.01, Fig. 6E) but also attenuated NMDAR1 expression in glutamate-induced PC12 cells (*P* < 0.05, Fig. 6F). These findings were consistent with the Western blot results. Collectively, SZJN formula might repress glutamate toxicity partially by modulating the expression of NMDAR1 and CME molecules.

### CME inhibitors partly blocked SZJN formula-induced neuroprotective effect

**Fig. 7:**
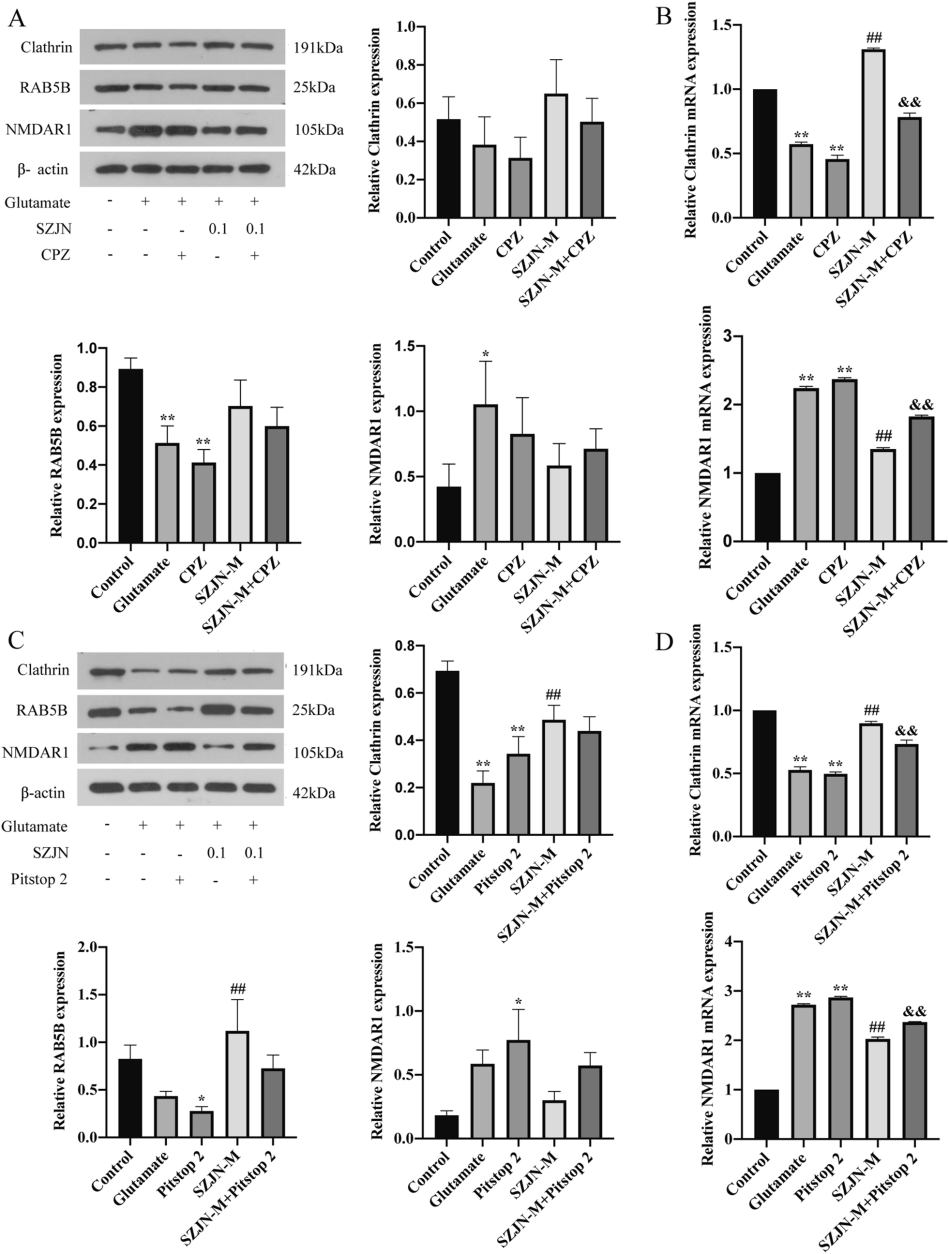
The promotion of SZJN formula on the process of CME could be blocked by CME inhibitors. **A** Western blot was performed to detect the protein levels of clathrin, RAB5B, and NMDAR1 in glutamate-injured PC12 cells after treatment with CPZ or SZJN formula (0.1 mg/mL). β-Actin was used as the internal control. **B** qRT-PCR analyzed clathrin mRNA and NMDAR1 mRNA expression levels in glutamate-injured PC12 cells after treatment with CPZ or SZJN formula (0.1 mg/mL). **C** Western blot was performed to detect the protein levels of clathrin, RAB5B, and NMDAR1 in glutamate-injured PC12 cells after treatment with Pitstop 2 or SZJN formula (0.1 mg/mL). β-Actin was used as the internal control. **D** qRT-PCR analyzed clathrin mRNA and NMDAR1 mRNA expression levels in glutamate-injured PC12 cells after treatment with Pitstop 2 or SZJN formula (0.1 mg/mL). Values were expressed as means ± SD (n = 3). ^*^P < 0.05, ^**^P < 0.01 as compared with the control group. ^#^*P* < 0.05, ^##^*P* < 0.01 as compared with the glutamate group. ^&^*P* < 0.05, ^&&^*P* < 0.01 as compared with the SZJN-M group

To explore whether neuroprotective effect induced by SZJN formula was dependent on the activation of CME process, we used the clathrin-mediated endocytosis inhibitors CPZ and Pitstop 2 in glutamate-treated PC12 cells. When CPZ or Pitstop 2-treated PC12 cells were incubated with glutamate, the mRNA and protein expression of clathrin and RAB5B was decreased and NMDAR1 was increased to different extents, compared to that in untreated PC12 cells (*P* < 0.05, Fig. 7). Besides, the protein level of clathrin and RAB5B were downregulated and NMDAR1 were upregulated in the SZJN-M group treated with endocytosis inhibitors, albeit not significantly (*P* > 0.05, Fig. 7A and C). The SZJN-M group treated with endocytosis inhibitors experienced a significant decrease in the clathrin mRNA level and increase in the NMDAR1 mRNA level compared to the SZJN-M group without endocytosis inhibitors (*P* < 0.01, Fig. 7B and D). This result indicates that the administration of CPZ or Pitstop 2 partly inhibited the SZJN-induced protective effect in injured PC12 cells.

## Discussion

In the present study, we demonstrated that SZJN formula could reduce the neurons damage, promote cell proliferation and attenuate cognitive deficits both in VD rats and cell models. Moreover, SZJN formula down-regulated NMDAR1 expression level, as well as activated the key molecules of the endocytosis process such as clathrin and RAB5B. The process of endocytosis as a possible mechanism was tested by exposing cells to two endocytosis inhibitors, suggesting that the CME of NMDA receptors may involve in the inhibitory effects of SZJN formula on glutamate excitotoxicity, which might account for a prosurvival effect and cognitive improvement of SZJN formula.

Cerebral ischemia hypoperfusion injury could impair the ability of working memory and spatial learning and memory. Emerging evidence has suggested that SZJN formula administration significantly recovered the impaired spatial memory induced by hypoxia and ameliorated the hippocampus-dependent memory dysfunction induced by cyclophosphamide and scopolamine [32, 37]. However, it is still unknown whether SZJN formula could attenuate cognitive deficits in VD rats. In this study, SZJN formula and memantine hydrochloride administration reduced the escape latency as well as the total distance and prolonged the time in the target quadrant, suggesting that SZJN formula treatment inhibited the spatial memory impairment induced by 2VO. These results were consistent with previous studies and indicated that SZJN formula showed the cognition-enhancing effects on VD.

Numerous studies have demonstrated that cerebral ischemia–reperfusion results in hippocampal injury, which is a contributor to cognition decline [38, 39]. The Nissl body is a normal structure in the cytoplasm of neurons and has the function of protein synthesis in neurons [40]. Nissl body fragmentation could be investigated when injured by cerebral ischemia. Our previous researches suggested that SZJN formula is effective in inhibiting apoptosis, enhancing the concentrations of neurotransmitters, repairing mitochondrial function, and improving cerebral blood flow [31, 37]. In this study, our results showed 2VO injury caused obvious pathological abnormalities manifested as loosed arranged neurons, destroyed Nissl bodies, cell degeneration, or dark color staining in the hippocampal CA1 region. However, SZJN formula significantly reversed neuron damage. Moreover, SZJN formula inhibited the glutamate-induced PC12 cell damage and death. The findings in the present study were consistent with previous studies, suggesting SZJN formula has a neuroprotective effect against vascular dementia.

The pathogenesis of VD includes various aspects, such as neuronal apoptosis, oxidative stress, and neurotransmitter abnormities. In particular, many studies have mentioned that glutamate neurotoxicity cannot be ignored in the development of cognitive impairment with VD [41, 42]. Studies have shown that neuronal death induced by cerebral ischemia and reperfusion is mainly associated with the high concentrations of glutamate and excessive activation of NMDAR [43, 44]. Glutamate overaccumulation leads to glutamatergic neurotransmitter dysfunction, signal transmission disorder, and oxidative stress injury. During the early stages of ischemia and hypoxia, NMDA receptors expression in the hippocampal CA1 region of VD rats increases, which aggravates the excitotoxic effect of glutamate. Excessive glutamate continues to stimulate postsynaptic NMDA receptors, resulting in over activation of NMDA receptors and excessive Ca^2+^ influx, ultimately resulting in calcium overload, neurons injury, and cognitive dysfunction. A previous study has demonstrated that reducing glutamate release and inhibiting NMDA receptors activity could inhibit Ca^2+^ influx and alleviate the cytotoxic injury [45]. Besides, as the most important subunit structure of NMDA receptors, NMDAR1 is responsible for synaptic transmission and plasticity. A growing number of studies have suggested that the loss of NMDAR1 or the mutations in genes that encode NMDAR1 results in the total loss of NMDAR function, is associated with many neuropsychiatric disorders [46–48]. Mutant NMDAR1 subunit has reduced glycine sensitivity, which is correlated with reduced forward trafficking of NMDARs to the cell surface [49]. In the present study, we found that SZJN formula could prominently reduce the protein and mRNA expression levels of NMDAR1. These results suggested that SZJN formula may decline the overaccumulation of glutamate to alleviate glutamate neurotoxicity.

The over activation of NMDA receptors could not only lead to the Ca^2+^ influx, but also affect the internalization mediated by clathrin. A study investigated that the NMDA-evoked Ca^2+^ influx could affect the expression of clathrin, the synaptic protein that plays an important role in synaptic vesicular formation and transport of glutamate and other excitatory amino acids [50]. Clathrin, mediating the classical endocytosis, is widely involved in signal transduction of various physiological activities, including nutrient absorption, cell growth and differentiation, synaptic transmission, and immune response. Binding to NMDAR1 receptors of postsynaptic membrane needs the mediation of clathrin and the internalization of NMDA receptors also relies on clathrin [51, 52]. Several studies have reported that the process of endocytosis is critical for NMDAR mediated excitotoxicity. Excessive activation of NMDAR triggers in increased endocytosis, which may be an important step in NMDAR mediated excitotoxic neuronal death [53, 54]. The apoptosis induced by over activation of NMDAR In Drosophila melanogaster was inhibited by dynamin, which could block the endocytosis mediated by clathrin [55]. Besides, clathrin is pivotal for the fusion of synaptic vesicles and presynaptic membranes. Once synaptic vesicles lose their endocytosis function, the area around the presynaptic membranes increase. This will result in a gradual decrease in lateral membrane tension, which consequently cause neurons to lose their normal synaptic functions [56, 57]. Studies have shown that several disease-related genes can damage and interfere with vesicle endocytosis, which then results in decreased levels of endocytosis-related proteins such as clathrin, AP-2, and dynamin [58–60]. RAB5B is a small ATPase molecule that has been shown as a regulator in the early endocytic pathway of cell surface NMDARs [10]. It has been investigated that the internalization of NMDAR1 was accompanied by increased RAB5B expression. Moreover, other studies have shown that the clathrin-mediated endocytosis is also involved in amyloid-β and a-synuclein clearance or transmission. For instance, a-synuclein oligomerization promoted NMDAR1 subunit internalization [61]. Amyloid-β could decrease the surface expression of NMDA receptors by promoting endocytosis of receptor proteins [62]. Our study showed that SZJN formula could significantly up-regulate clathrin and RAB5B expression, and we speculated that was associated with the significant reduction of surface NMDAR1 expression, which may be closely involved in promoting endocytosis of NMDARs and attenuating toxic glutamate accumulation.

The endocytosis of neuronal synaptic vesicles mediated by clathrin involves a variety of complex procedures and acts as an important pathological process of VD. It plays an important role in transcellular transport, information transmission and signal transduction. The inhibition of endocytosis will lead to the accumulation of glutamate among neurons, resulting in synaptic loss and dysfunction. CPZ and Pitstop 2 are clathrin-mediated endocytic pathway inhibitors, which means that they can inhibit the function of clathrin and block the assembly of clathrin coated pits at the plasma membrane [63, 64]. Our results revealed that both CPZ and Pitstop 2 could inhibit the process of CME to different extents and partly block SZJN formula-induced neuroprotective effect. Based on the clathrin mediated endocytosis, this study explored the neuroprotective effects of SZJN formula against VD, which is helpful to clarify the pathological mechanism of VD and how SZJN formula can make an affect. Moreover, this study provided an objective basis for the clinical use of SZJN formula. The results support SZJN formula as a potentially promising therapy for treatment of cognitive dysfunction in patients with VD. The endocytosis process is not only dependent on the endocytosis proteins such as clathrin, but also closely related to a variety of proteins, including adapter proteins and accessory proteins. Based on these results, we could further explore the relationships among these proteins in the future, which will help to further clarify the role of the process of CME in the pathogenesis of VD. In addition, studying the active components of SZJN formula acting on the endocytosis pathway will be of significance to explore new targets, develop new drugs and clinical research for the treatment of VD.

The present study uncovered that SZJN formula could attenuate neurons damage and cognitive deficits in VD, and the role of SZJN formula in the reducing glutamate cytotoxicity may be related to the regulation of the key molecules of clathrin mediated endocytosis of NMDAR receptors. However, there are several limitations needing to be improved in our study. Firstly, we did not measure the level of glutamate in vivo, as well as the expression of clathrin combined with NMDA receptors on the membrane. Secondly, we only found that SZJN formula regulated these classical endocytosis molecules, but whether SZJN formula could regulate other key targets through this pathway has not been verified. In the future, more cell types need be employed to validate the study results. It is indispensable to use primary cultured neurons or other cell lines to verify the micro-mechanism of SZJN formula on clathrin-mediated endocytosis. In addition, more studies can be carried out using advanced imaging technology to explore the specific mechanism of SZJN formula on clathrin mediated NMDA receptors endocytosis in VD.

## Conclusions

In summary, we demonstrated that SZJN formula significantly attenuated neuronal damage, glutamate cytotoxicity and cognitive deficits in VD, which was probably related to the regulation of the glutamate synaptic vesicle transport. Enhancement of clathrin and RAB5B expression may be involved in the internalization of NMDA receptors mediated by CME after SZJN formula treatment. These results suggest that SZJN formula may be a potential therapeutic agent for the prevention and treatment of VD. Additional experimental and clinical studies will be required to assess the potential for clinical application of SZJN formula.

## Supplementary Information

**Additional file 1: Fig. S1.** Information of herbs in SZJN formula. (A) Representative images of herbs in SZJN formula. (B) List of herbal names in SZJN formula.

**Additional file 2: Fig. S2.** High Performance Liquid Chromatography (A) and infrared spectrum (B) profiles of standards and SZJN formula granules.

**Additional file 3: Fig. S3.** The Initial screening on the concentrations of SZJN formula and glutamate using a CCK8 assay. (A) SZJN formula. (B) Glutamate.

**Additional file 4: Table S1.** Antibodies used for immunohistochemistry, immunofluorescence, and western blot. **Table S2.** Primer sequences for quantitative real-time PCR.

## Data Availability

The data used to support the current study are available from the corresponding author on reasonable request.

## Abbreviations

SZJN formula: Shenzhi Jiannao formula
VD: Vascular dementia
NMDAR1: *N*-methyl-d-aspartic acid receptor 1
CME: Clathrin-mediated endocytosis
HPLC: High-performance liquid chromatography
IS: Infrared spectrum
PVDF: Polyvinylidene fluoride
