# Supplementary figures and images for: Shenzhi Jiannao formula ameliorates vascular dementia in vivo and in vitro by inhibition glutamate neurotoxicity via promoting clathrin-mediated endocytosis
Source: Chin Med. 2021 Jul 28;16:65. doi: 10.1186/s13020-021-00477-4 (PMC8317332; doi:10.1186/s13020-021-00477-4)

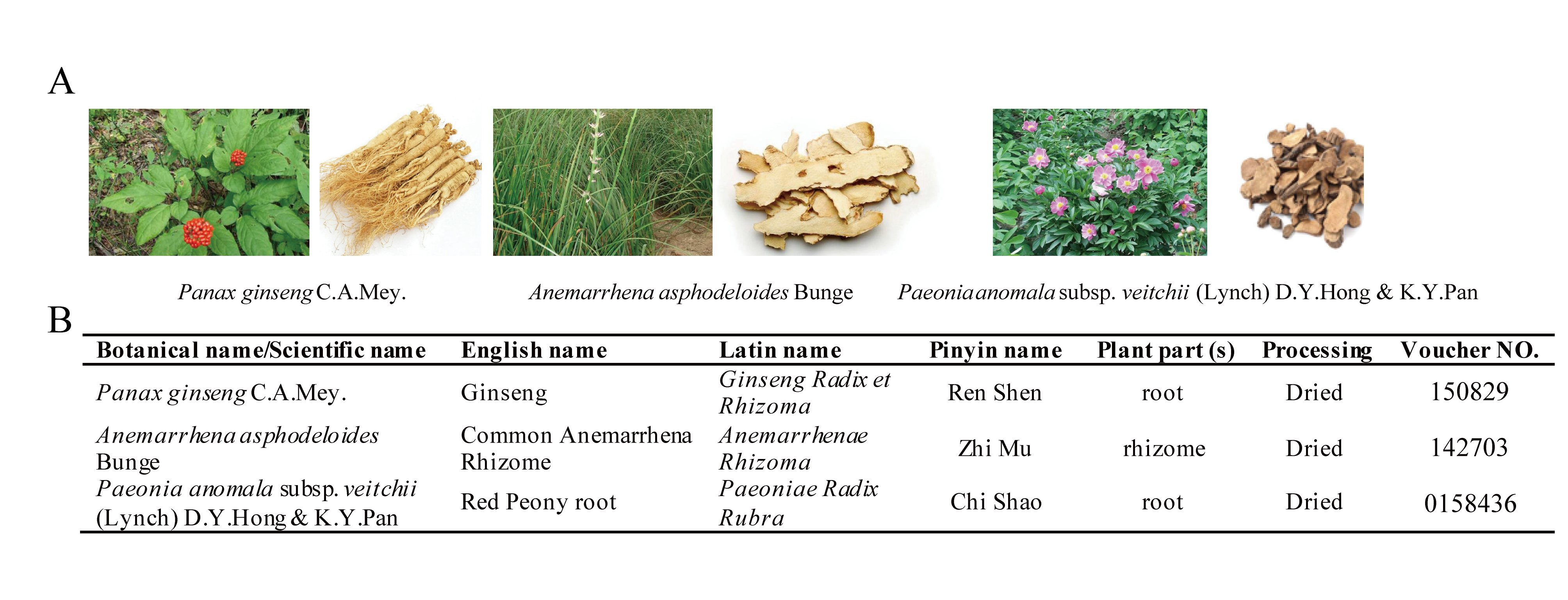

Supplement: Supplementary file 1 — Additional file 1: Fig. S1. Information of herbs in SZJN formula. (A) Representative images of herbs in SZJN formula. (B) List of herbal names in SZJN formula. [file 13020_2021_477_MOESM1_ESM.tif]

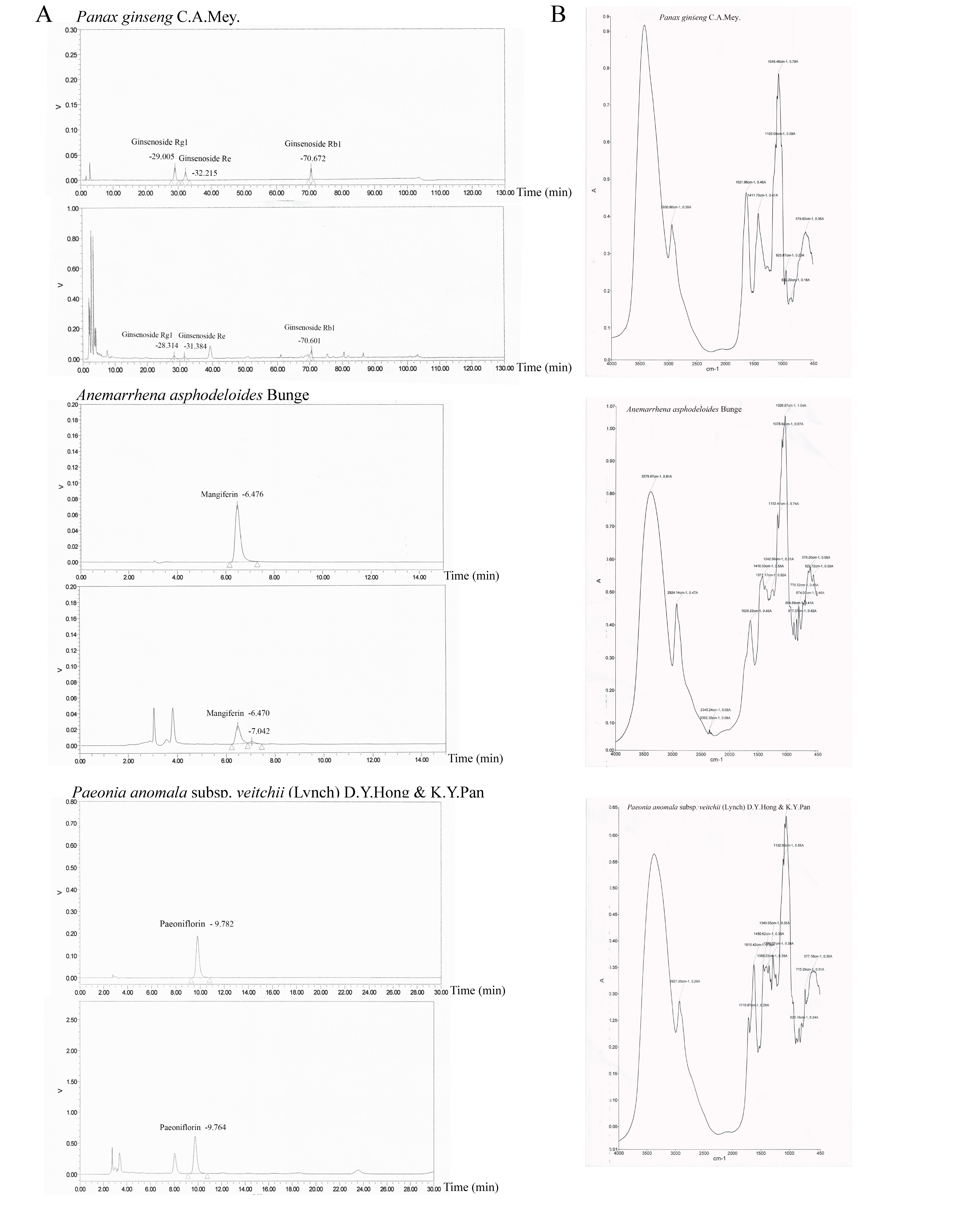

Supplement: Supplementary file 2 — Additional file 2: Fig. S2. High Performance Liquid Chromatography (A) and infrared spectrum (B) profiles of standards and SZJN formula granules. [file 13020_2021_477_MOESM2_ESM.tif]

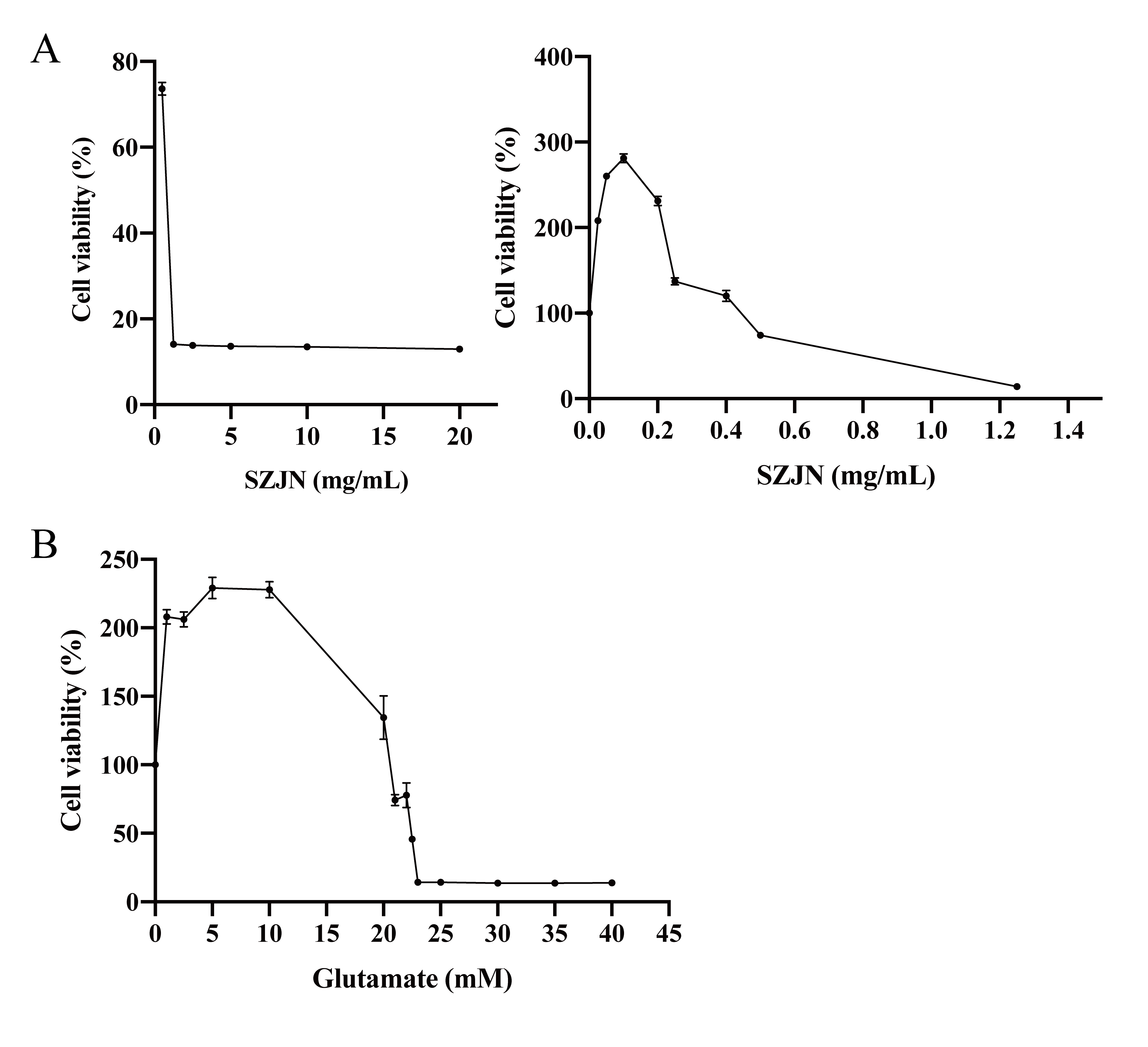

Supplement: Supplementary file 3 — Additional file 3: Fig. S3. The Initial screening on the concentrations of SZJN formula and glutamate using a CCK8 assay. (A) SZJN formula. (B) Glutamate. [file 13020_2021_477_MOESM3_ESM.tif]
