# Supplementary material for: Shenzhi Jiannao formula ameliorates vascular dementia in vivo and in vitro by inhibition glutamate neurotoxicity via promoting clathrin-mediated endocytosis
Source: Chin Med. 2021 Jul 28;16:65. doi: 10.1186/s13020-021-00477-4 (PMC8317332; doi:10.1186/s13020-021-00477-4)
Supplement: Supplementary file 4 — Additional file 4: Table S1. Antibodies used for immunohistochemistry, immunofluorescence, and western blot. Table S2. Primer sequences for quantitative real-time PCR. [file 13020_2021_477_MOESM4_ESM.docx]

**Shenzhi Jiannao formula ameliorates vascular dementia *in vivo* and *in vitro* by inhibition glutamate neurotoxicity via promoting clathrin-mediated endocytosis**

**Supplementary Tables**

**Table S1. Antibodies used for immunohistochemistry, immunofluorescence, and Western blot.**

| **Experiments** | **Name** | **Company** | **Number** | **Dilution** | **Description** |
| --- | --- | --- | --- | --- | --- |
| Immunohistochemistry | Clathrin | Abcam | ab172958 | 1:50 | Rabbit mAb |
|  | NMDAR1 | Abcam | ab68144 | 1:100 | Rabbit mAb |
|  | RAB5B | Proteintech | 17605-1-AP | 1:50 | Rabbit pAb |
|  | Secondary antibody | Applygen Technologies | C1309 | 1:200 | HRP-labeled Goat Anti-Rabbit IgG |
| Immunofluorescence | Clathrin | Proteintech | 66487-1-Ig | 1:500 | Mouse mAb |
|  | NMDAR1 | Abcam | Ab17345 | 1:1000 | Rabbit pAb |
|  | RAB5B | Thermo Fisher Scientific | PA5-44574 | 1:5000 | Rabbit pAb |
|  | Alexa Fluor® 488 | Abcam | Ab150113 | 1:400 | Goat Anti-Mouse IgG H&L |
|  | Alexa Fluor® 594 | Abcam | Ab150080 | 1:500 | Goat Anti-Rabbit IgG H&L |
| Western blot | Clathrin | Abcam | ab172958 | 1:80000 | Rabbit mAb |
|  | NMDAR1 | Abcam | ab68144 | 1:2000 | Rabbit mAb |
|  | RAB5B | Proteintech | 17605-1-AP | 1:500 | Rabbit pAb |
|  | Secondary antibody | Applygen Technologies | C1309 | 1:2000 | HRP-labeled Goat Anti-Rabbit IgG |
|  | β-actin | Proteintech | 20536-1-AP | 1:50000 | Rabbit pAb |

**Table S2. Primer sequence for quantitative real- time PCR.**

| Genes (Rats) | Forward primer (5^’^ to 3^’^) | Reverse primer (5^’^ to 3^’^) | Length (bp) |
| --- | --- | --- | --- |
| Clathrin | CTCTGAGAATGGCTGTGCGGAAC | TCGACGGATGGTATCTGGAGTACG | 158 |
| NMDAR1 | ATGTGGTGGCTGTGATGCTGTAC | TTCCTCCTCCTCCTCACTGTTCAC | 83 |
| β-actin | GCAGTTGGTTGGAGCAA | ATGCCGTGGATACTTGGA | 114 |

Primers were synthesized by the Sangon Biotech Company (Shanghai, China).

**Supporting Figure Legends**

**Figure S1. Information of herbs in SZJN formula. (**A) Representative images of herbs in SZJN formula. (B) List of herbal names in SZJN formula.

**Figure S2. High Performance Liquid Chromatography (A) and infrared spectrum (B) profiles of standards and SZJN formula granules.**

**Supplementary Figure 3. The Initial screening on the concentrations of SZJN formula and glutamate using a CCK-8 assay.** (A) SZJN formula. (B) glutamate.

**Table S1. Antibodies used for immunohistochemistry, immunofluorescence, and Western blot.**

**Supplementary Table 2. Primer sequences for quantitative real- time PCR.**

**Supporting Materials and Methods**

**Chemical Constituents in SZJN formula using high-performance liquid chromatography**

**Materials and Reagents**

Acetonitrile and methanol as high-performance liquid chromatography grade were purchased from Thermo Scientific (USA). Formic acid as high-performance liquid chromatography grade was purchased from Aladdin Biochemical Technology Co., Ltd. (Shanghai, China). Phosphoric acid as analytical grade was purchased from Beijing chemical plant (Beijing, China). Milli-Q water was prepared using a Milli-Q system (Millipore, MA, USA). Ginsenoside Rg1, ginsenoside Re, ginsenoside Rb1, Mangiferin, and paeoniflorin (the purities of all standards were higher than 98% by high-performance liquid chromatography analysis) were purchased from Solarbio Biotech Co., Ltd. (Beijing, China).

**Sample preparation**

SZJN formula granules, 0.5g, were weighed and transferred into a 50 mL volumetric flask. Methanol was added and ultrasonic for 30 min. The volume was fixed with methanol to the scale, filtered through 0.22 μm organic filter membrane, and tested on the machine.

**High performance liquid chromatography (HPLC) of 5 compounds in the SZJNF granules**

The quantifications of 5 compounds in the SZJNF granules were performed by an E2695-Waters High performance liquid chromatography (HPLC) Class system (Waters Corp., Milford, USA) coupled with a Zorbax AQ C18 column (4.6 mm × 250 mm, 5 μ m, Agilent, USA) maintained at 40°C. Elution was performed with a mobile phase of A (0.1% formic acid in water) and B (0.2% phosphoric acid in acetonitrile). The flowrate was 0.2 ml/min, and the injection volume was 10 μl. The analytes were monitored at the UV wavelength of 203 nm (*Panax ginseng* C.A.Mey., Ren Shen), 258 nm (*Anemarrhena asphodeloides* Bunge, Zhi Mu), and 230 nm (*Paeonia anomala* subsp. *veitchii* (Lynch) D.Y.Hong & K.Y.Pan, Chi Shao). The total run time was 130 minutes (Ren Shen), 20 minutes (Zhi Mu), and 30 minutes (Chi Shao).
